# Microbiological diversity among patients with Lemierre syndrome and clinical implications: an individual patient-level analysis

**DOI:** 10.1007/s15010-025-02489-w

**Published:** 2025-02-15

**Authors:** Maurus Frehner, Riccardo M. Fumagalli, Silvio D. Brugger, Silvia Cardi, Filippo Catalani, Alice Trinchero, Alessandro Pecci, Nils Kucher, Luca Valerio, Stefano Barco

**Affiliations:** 1 Department of Angiology, University Hospital Zurich, Zurich, Switzerland; 2 University of Zurich, Zurich, Switzerland; 3 Department of Infectious Diseases and Hospital Epidemiology, University Hospital Zurich, Zurich, Switzerland; 4 Department of Biomedical Sciences, Humanitas University, Pieve Emanuele, Milan, Italy; 5 IRCCS Humanitas Research Hospital, Rozzano, Milan, Italy; 6 Department of Internal Medicine, Regional Hospital of Bellinzona and Valli, Ente Ospedaliero Cantonale, Bellinzona, Switzerland; 7 Department of Cardiology, University Medical Center Mainz, Mainz, Germany; 8 Center for Thrombosis and Hemostasis, University Medical Center Mainz, Langenbeckstrasse 1, 55131 Mainz, Germany; 9 Department of Medical Oncology and Haematology, University of Zurich and University Hospital Zurich, Zurich, Switzerland; 10 Department of Internal Medicine, IRCCS Policlinico San Matteo Foundation, University of Pavia, Pavia, Italy

**Keywords:** *Fusobacterium*, Infectious disease, Lemierre syndrome, Septic embolism, *Staphylococcus*, Thrombosis

## Abstract

**Purpose:**

Lemierre syndrome is a rare condition traditionally defined by bacterial infection of the head/neck region, local thrombophlebitis, and septic embolism. Although in most cases *Fusobacterium necrophorum* is isolated, it is questionable whether the presence of this microbe is mandatory for diagnosis. In this study, we investigated microorganisms isolated in cases of Lemierre syndrome and their association with demographical and clinical features.

**Methods:**

We conducted an analysis of individual patient data from 712 patients diagnosed with Lemierre syndrome. Demographics, clinical presentation, treatment strategies, and outcomes according to different pathogens were evaluated.

**Results:**

Among a total of 712 patients, in 574 cases bacterial growth was detected. In 415 patients *Fusobacterium* spp. was isolated, in 108 either *Streptococcus* spp. or *Staphylococcus* spp., and in 51 other bacteria. Patients with different bacteria differed markedly in age, site of preceding infections, clinical presentation, and treatment. *Fusobacterium* spp. was typically isolated in younger patients (69% of patients aged 16 to 30 years) while *Streptococcus* spp. and *Staphylococcus* spp. were more prevalent in older subjects (30% of patients aged over 45 years). Of all cases with *Fusobacterium* spp., 63% had a thrombosis of the internal jugular vein and 91% septic embolism, compared with 94% and 69%, respectively, in cases with *Streptococcus* spp. or *Staphylococcus* spp.

**Conclusion:**

In contrast to the available literature, our study suggests that Lemierre syndrome may be caused by multiple bacterial species, and that the clinical presentation and course may vary according to the specific bacterial species involved.

**Supplementary Information:**

The online version contains supplementary material available at 10.1007/s15010-025-02489-w.

## Introduction

Lemierre syndrome, previously also known as post-anginal septicaemia or necrobacillosis, typically affects adolescents and young adults and is characterized by internal jugular vein thrombophlebitis, bacteremia, and metastatic septic emboli secondary to acute pharyngeal infections. The estimated incidence rates vary between one and four cases per million general population-years, with figures ten times higher in the affected age group [1, 2]. If not adequately treated, Lemierre syndrome can be fatal or lead to long-lasting sequelae. With modern antibiotic therapies, the estimated case fatality rate, despite the young and otherwise healthy patient population, ranges from 4 to 9% [2, 3].

A universally accepted definition and diagnostic criteria for this disease have not been established, possibly because of the lack of large patient populations with consistent data [4, 5].The diagnosis of Lemierre syndrome traditionally relies on three criteria: oropharyngeal infection, dissemination as septic thrombosis of the internal jugular vein or distant embolism, and identification of *Fusobacterium necrophorum* subsp. *funduliforme*. The site of primary infection usually includes the oropharynx, but the initial focus may originate from other sites within the entire head/neck district. The criterion of dissemination is normally met with the evidence of jugular vein thrombosis, septic emboli or bacteremia.

Whether Lemierre syndrome should be restricted to a single bacterium is also a topic of discussion [6–8]. *Fusobacterium* spp., notably *F. necrophorum*, is traditionally considered the single etiologic agent of the disease. Two major subspecies exist: (i) subsp. *necrophorum* (biotype A), which is mainly found in animals and (ii) subsp. *funduliforme* (biotype B), which is commonly found in the human oral cavity and intestinal tract. Hereafter, “*F. necrophorum*” will denote the subspecies *funduliforme*. *Fusobacterium spp.* specifically. *F. necrophorum* carries several virulence factors that might contribute to the development of Lemierre syndrome; these include leukotoxins, endotoxins, and aggregation factors such as hemagglutinin [9–12]. However, several arguments exist for the possibility that other pathogens may cause Lemierre syndrome either in combination with *Fusobacterium spp.* or in its complete absence. First, previous series frequently report polymicrobial blood cultures in Lemierre syndrome. Whether this simply reflects co-infection is uncertain, as other organisms are much more commonly found in *Fusobacterium*-negative cases than in *Fusobacterium*-positive cases (while a similar prevalence would be expected if the explanation was co-infection) [13]. Second, several of these bacteria – especially *Staphylococcus aureus* and *Streptococcus* spp. – have themselves known thrombogenic properties and their infections have been reported to manifest as clinical syndromes clinically indistinguishable from Lemierre syndrome [6, 8, 14–18]. Third, in vitro studies have shown some of these bacteria to act synergistically with *Fusobacterium* spp., with the presence of one enhancing the infectivity of the other [19–21].

We studied the presentation and clinical course of Lemierre syndrome with respect to the underlying microbiological findings in a large population of patients with Lemierre syndrome.

## Patients and methods

As described previously, we collected patient-level data searching for all studies reporting cases of Lemierre syndrome published between January 2000 and August 2017 in MEDLINE, Embase, and Cochrane Library without language or study design restrictions [3]. Results from grey literature, which comprises all relevant research results not reported in the traditional academic publishing and distribution channels, were also included. We followed the Preferred Reporting Items for Systematic Review and Meta-Analyses of individual patient data (PRISMA-IPD) statement to identify data for extraction, which was adapted to the current setting. Our study protocol is registered in the International Prospective Register of Systematic Reviews PROSPERO (CRD42016052572). To be eligible, cases needed to meet the following criteria: (i) the primary site of bacterial infection being reported to be located in the head/neck area and (ii) the objective confirmation of local thrombotic complications or septic embolism. This corresponded to an “extended” definition of Lemierre syndrome. This approach, while encompassing cases that meet the oldest and most restrictive definition of Lemierre syndrome, also included: (i) patients with primary infectious foci localized beyond the oropharyngeal region, involving other anatomical structures within the head and neck (e.g., facial and external jugular veins, cerebral sinuses, or carotid arteries), and (ii) patients with bacteria other than *Fusobacterium* spp., which are now increasingly recognized as potential etiological agents associated with this syndrome [4, 22–25]. Only those patients with information on microbiological status (including those with negative blood cultures) were included in this analysis and subsequently stratified according to the microbiological findings in the following groups: (i) patients with a culture positive for *Fusobacterium* spp. (obligate anaerobes), (ii) patients with a culture positive for *Streptococcus* spp. and/or *Staphylococcus* spp. (aerotolerant anaerobes), (iii) patients with a culture positive for other bacteria, and (iv) patients with negative cultures. Positive cultures were defined as bacterial growth in culture of a normally sterile fluid. If *Fusobacterium* spp. was isolated simultaneously with other bacteria, the patients were still classified in the *Fusobacterium* spp. group. If *Streptococcus* spp. or *Staphylococcus* spp. were isolated with any other bacteria than *Fusobacterium* spp., cases would be classified as *Streptococcus* spp./*Staphylococcus* spp. Information on bacterial genus and species was extracted to the highest level of detail reported in the patient’s medical documentation or provided by the authors of the case series and case reports. The specific microbiological techniques used, such as culture conditions (i.e., aerobe, anaerobe, time of incubation), identification techniques (e.g., MALDI-TOF MS, 16 S rRNA gene sequencing, or gas chromatography mass spectrometry (GC-MS)), were not reported with a consistent level of detail.

Baseline characteristics, including age, sex, type of primary head or neck infection, and clinical signs and symptoms, were described. We considered the following clinical outcomes as early complications: (i) development of new or recurring venous thromboembolism, (ii) new or worsening peripheral septic lesions, (iii) major bleeding, and (iv) death from any cause within 30 days of the diagnosis of Lemierre syndrome or during hospital stay. New or worsening peripheral septic lesions referred to the development of new multiple sub-segmental lung emboli, abscesses or empyemas, arterial ischemia, septic arthritis, or an objective worsening of initial embolic manifestations after the diagnosis of Lemierre syndrome. Major bleeding was defined according to the criteria set by the International Society on Thrombosis and Haemostasis [26]. Long-term complications were defined as clinical sequelae observed at the time of discharge from the hospital or persisting during post-discharge follow-up, such as cranial nerve palsy, other neurological complications, or orthopedic or functional limitations.

We collected data on the use of antibiotics, anticoagulants, and surgical procedures for each patient. We only included cases that provided data on the clinical course beyond the initial presentation in the analysis of study outcomes.

Categorical variables were summarized as counts and percentages, while continuous variables were presented as either medians and interquartile ranges (Q1–Q3) or means and standard deviations (SD), depending on their distribution. Standard descriptive statistical methods and graphical techniques were applied to analyze baseline characteristics, therapeutic interventions, and clinical outcomes, stratified by culture results as described previously. To account for the potential impact of contaminant species and the heterogeneity of the microbiological testing methods, a sensitivity analysis was conducted, recalculating the distribution of culture results, baseline characteristics, and clinical outcomes across patients in whom cultures of other normally sterile body sites (suppurations, exudates, CSF) were carried out in addition to blood cultures with those in whom blood cultures alone were carried out.

Data management and analysis was conducted with R version 3.5.1 (The R Foundation for Statistical Computing, 2018).

## Results

### Study population

Of a total of 712 patients, 574 (81%) were reported to have at least one positive culture during the index hospitalization. Baseline characteristics stratified by isolated pathogens are illustrated in Table 1. Of the patients, 295 (41%) were women; median age at presentation was 21 years (Q1-Q3: 17–33). Approximately half of the patients (*n* = 352) were treated in Europe, with most of the remaining treated in Northern America (*n* = 221) and in the Asia-Pacific Region (*n* = 129). A total of 373 (52%) of the cases were reported from academic medical centres or university hospitals.

**Table 1:** Baseline characteristics of the study population according to microbiological findings

	*Fusobcterium* spp. (*n* = 415)	*Streptococcus/Staphylococcus* spp. (*n* = 108)	Other (*n* = 51)	Negative Culture (*n* = 138)	Total (*n* = 712)
Women	164 (40%)	43 (40%)	25 (49%)	63 (46%)	295 (41%)
Median age [Q1, Q3]	20 (16, 27)	25 (16, 49)	32 (19, 54)	24 (18, 43)	21 (17, 33)
Cancer	3 (1%)	4 (4%)	1 (2%)	4 (3%)	12 (2%)
**Site of Infection**					
Oropharyngeal Infection	333 (80%)	62 (57%)	22 (43%)	103 (75%)	520 (73%)
LRT Infection	214 (52%)	36 (33%)	21 (41%)	59 (43%)	330 (46%)
Neck Infection	136 (33%)	52 (48%)	23 (45%)	76 (55%)	287 (40%)
Ear Infection	45 (11%)	12 (11%)	11 (22%)	16 (12%)	84 (12%)
Dental Infection	26 (6%)	16 (15%)	8 (16%)	10 (7%)	60 (8%)
**Clinical Presentation**					
IJV Thrombosis	261 (63%)	101 (94%)	45 (88%)	119 (86%)	526 (74%)
Cerebral Vein Thrombosis	63 (15%)	38 (35%)	14 (28%)	28 (20%)	143 (20%)
EJV Thrombosis	20 (5%)	9 (8%)	3 (6%)	11 (8%)	43 (6%)
Septic Embolism	377 (91%)	74 (69%)	41 (80%)	90 (65%)	582 (82%)
Pulmonary Septic Embolism	326 (79%)	65 (60%)	33 (65%)	82 (59%)	506 (71%)
JBM Septic Embolism	72 (17%)	18 (17%)	7 (14%)	11 (8%)	108 (15%)
Intracranial Septic Embolism	52 (13%)	13 (12%)	5 (10%)	9 (7%)	79 (11%)
Liver Septic Embolism	9 (2%)	1 (1%)	2 (4%)	2 (1%)	14 (2%)

LRT = lower respiratory tract, IJV = internal jugular vein, EJV = external jugular vein, JBM = joints, bone or muscle

### Overall distribution of bacterial isolates

**Fig. 1:**
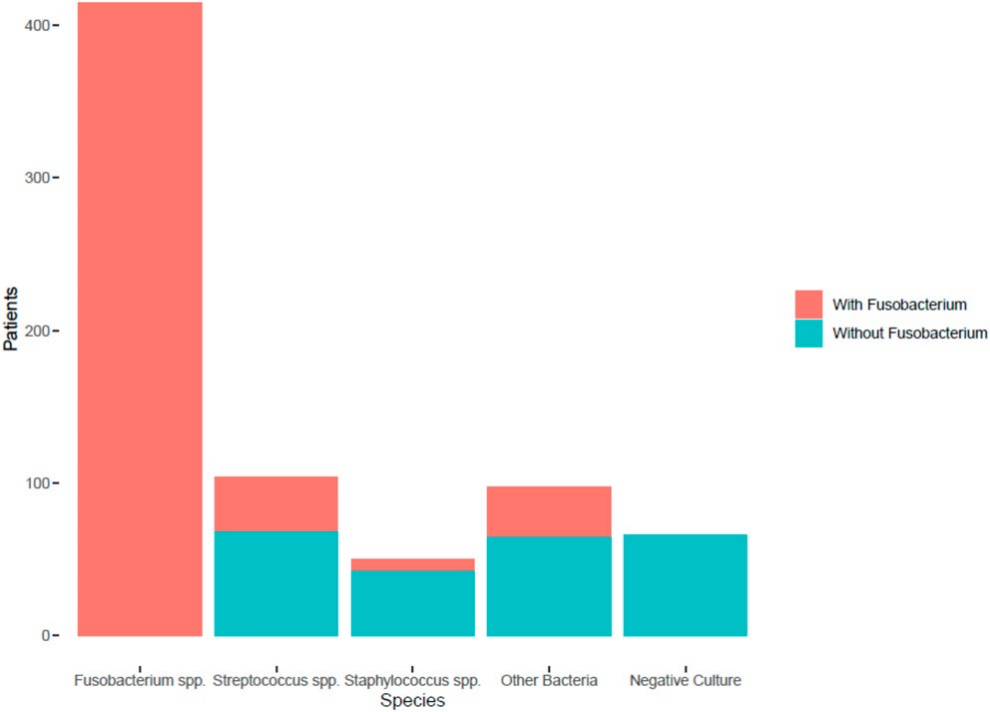
Bacterial species isolated in cases of Lemierre syndrome

Figure 1 displays the relationship between the defined subgroups. Obligate anaerobic bacteria were isolated in 448 (63%) patients. The most frequent isolated anaerobic organism was *Fusobacterium* spp. (*n* = 415; 58% of the total and 73% of positive). Among these, *Fusobacterium necrophorum* was isolated in 360 (51%) patients, *Fusobacterium nucleatum* in 30 (4%), and other unspecified Fusobacteria in 25 (3%) patients. Among the remaining 50 (7%) patients presenting with anaerobes, *Actinomyces* spp., *Bacteroides* spp., *Campylobacter* spp., *Enterococcus* spp., *Leptotrichia* spp., *Peptostreptococcus* spp., *Porphyromonas* spp., *Prevotella* spp., *Propionibacterium* spp., and *Veilonella* spp. were isolated. In 17 (2%) of these cases a *Fusobacterium* spp. was isolated concomitantly.

A total of 192 (27%) patients tested positive for at least one Firmicute (mainly *Streptococcus* spp. and *Staphylococcus* spp.): in 84 (12%) cases these co-existed with *Fusobacterium* spp., while in 108 (15%) patients *Streptococcus* spp. or *Staphylococcus* spp. alone were isolated. In 54 (8%) subjects, other bacteria were isolated, including *Acinetobacter* spp., *Arcanobacterium* spp., *Citrobacter* spp., *Corynebacterium* spp., *Escherichia coli*, *Eikenella* spp., *Enterobacter* spp., *Gemella* spp., *Haemophilus* spp., *Klebsiella* spp., *Micrococcus* spp., *Mycoplasma* spp., *Neisseria* spp., *Proteus* spp., *Pseudomonas* spp., *Serratia* spp., and *Trueperella* spp. Of these, 18 (2%) patients had a concomitant isolation of *Fusobacterium* spp.

### Clinical characteristics

**Fig. 2:**
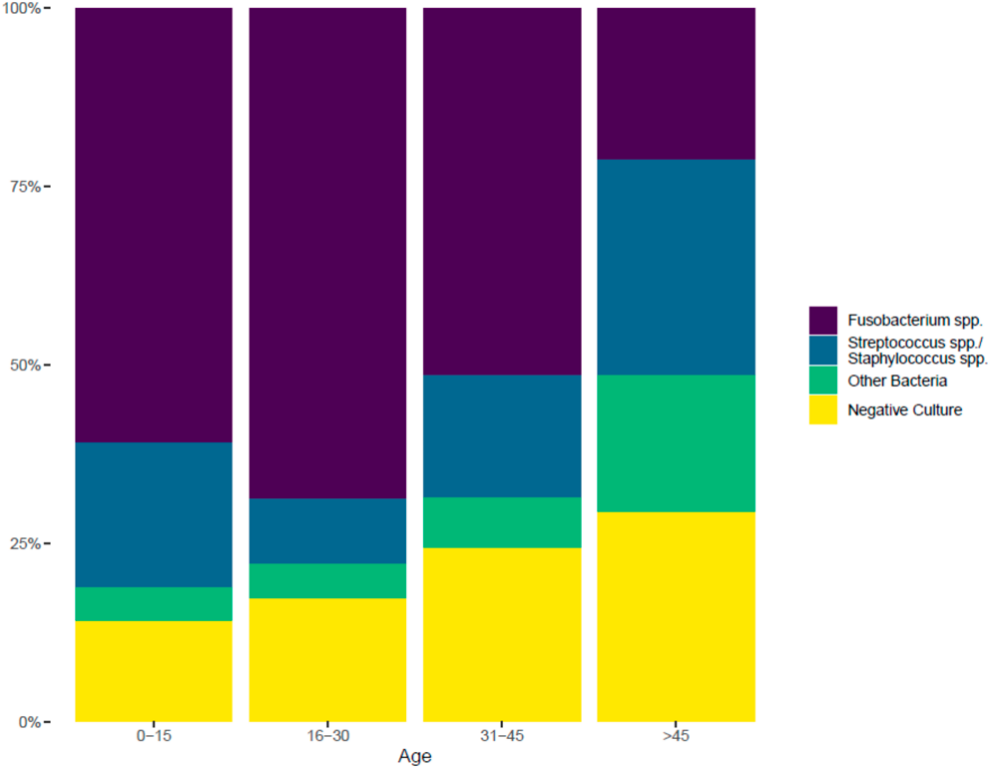
Age-dependent microbial patterns in patients with Lemierre syndrome

**Fig. 3:**
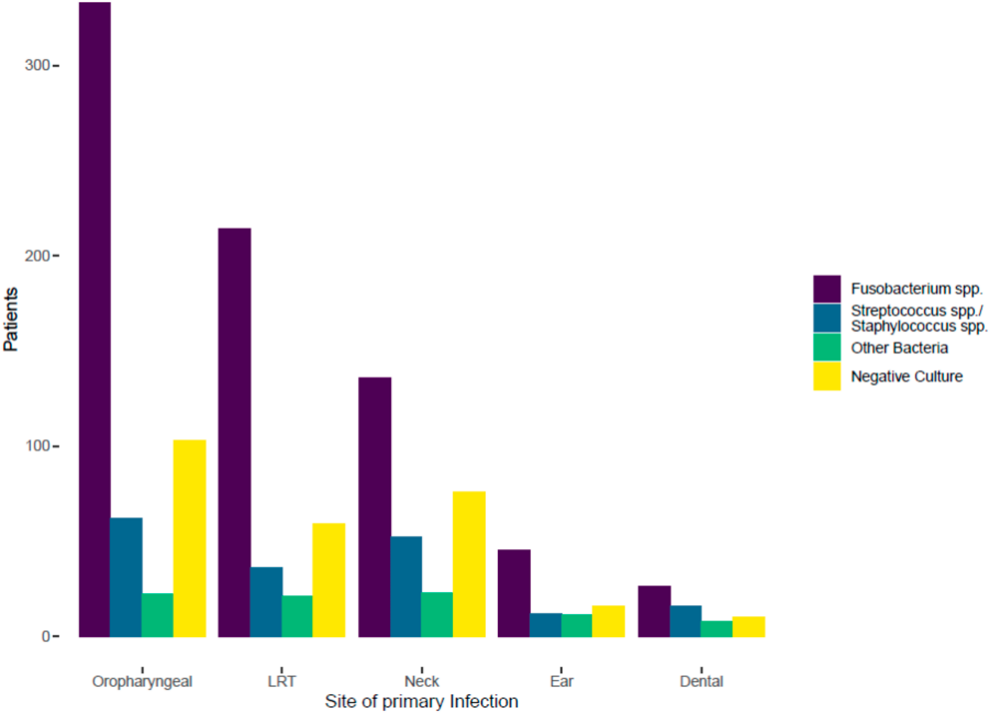
Distribution of bacterial species by site of primary infection at presentation. Legend: LRT lower respiratory tract

The prevalence of specific pathogens varied with age, as displayed in Fig. 2; Table 2. The vast majority of the patients was aged 16 to 30 years (*n* = 385, 54%), followed by younger patients of age 0 to 16 years (*n* = 128, 18%). *Fusobacterium* spp. was predominantly isolated among children and young adults, comprising 61% and 69% of all cases in the age groups of 0–15 and 16–30 years old, respectively. In contrast, only in 21% of the patients older than 45 years *Fusobacterium* spp. was isolated. The prevalence of *Streptococcus* spp. and *Staphylococcus* spp. appeared to be highest in patients below 16 years (20%) and in those older than 45 (30%). The entirety of all other bacteria was found in less than 10% of patients in all age groups except in the oldest patients’ group, where 19% of the subjects tested positive. The proportion of patients with negative cultures increased across age groups from 14% in those aged 0–15 years to 29% in those older than 45 years. No sex-related differences were observed for microbiological patterns. As shown in Table 1; Fig. 3, the presence of *Fusobacterium* spp. manifested in most cases with an oropharyngeal infection (*n* = 333, 80%), followed by lower respiratory tract infection (*n* = 214, 52%) or neck infection (*n* = 136, 33%). When *Streptococcus* spp. or *Staphylococcus* spp. were isolated, the presentation showed mostly oropharyngeal infections (*n* = 62, 57%) or neck infections (*n* = 52, 48%), followed by lower respiratory tract infection (*n* = 36, 33%). Conversely, the subgroup with all other bacteria showed an equal distribution among patients presenting with oropharyngeal (*n* = 22, 43%), neck (*n* = 23, 45%), or lower respiratory tract infection (*n* = 21, 41%). However, ear infections (*n* = 11, 22%) was a clinical manifestation in this subgroup about twice as often as in the subgroups *Fusobacterium* spp. (*n* = 45, 11%), *Streptococcus* spp. and *Staphylococcus* spp. (*n* = 12, 11%), and cases with negative culture (*n* = 16, 12%). The presence of the Epstein-Barr Virus was tested (either with polymerase chain reaction or by seroconversion) in 109 (15%) patients and found positive in 17 (2%).

**Table 2:** Microbiological findings according to patients’ age

	0–15 years (*n* = 128)	16–30 years (*n* = 385)	31–45 years (*n* = 99)	> 45 years (*n* = 99)	Total (*n* = 711)
*Fusobacterium* spp.	78 (61%)	265 (69%)	51 (52%)	21 (21%)	415 (58%)
*Streptococcus*/*Staphylococcus* spp.	26 (20%)	35 (9%)	17 (17%)	30 (30%)	108 (15%)
Other Bacteria	6 (5%)	19 (5%)	7 (7%)	19 (19%)	51 (7%)
Negative Culture	18 (14%)	66 (17%)	24 (24%)	29 (29%)	137 (19%)

The proportion of cases with initial internal jugular vein thrombosis was high across all patient groups (Table 1). However, this was the predominant thromboembolic manifestation at presentation among patients infected with either *Streptococcus* spp. or *Staphylococcus* spp. (*n* = 101, 94%), as well as among those with other bacterial infections (*n* = 45, 88%) or those with negative cultures (*n* = 119, 86%). Only in subjects with *Fusobacterium* spp., septic embolism, primarily pulmonary, emerged as the most frequent complication, affecting 377 (91%) patients. Internal jugular vein thrombosis was still a common complication, presenting in 261 (63%) patients. Besides, septic embolism was diagnosed in 74 (69%) patients with *Streptococcus* spp. or *Staphylococcus* spp., in 41 (80%) patients with other bacteria, and in 90 (65%) patients with negative blood cultures. Also, rates of cerebral vein thrombosis were lower in patients with isolation of *Fusobacterium* spp. (*n* = 63, 15%) than in those infected with *Streptococcus* spp. or *Staphylococcus* spp. and with other bacteria (*n* = 38, 35% and *n* = 14, 28%, respectively).

### Clinical course

An overview of the medical and interventional treatment received by the patients and stratified by bacterial group is provided in Table 3. The vast majority (*n* = 685, 96%) of patients received at least one antibiotic agent, five (1%) patients were managed without antibiotics, and in 22 (3%) cases no information about antimicrobial therapy was available. The most frequently used antibiotics were penicillins (*n* = 371, 52%), metronidazole (*n* = 318, 45%), cephalosporins (*n* = 279, 39%), clindamycin (*n* = 225, 32%), and carbapenems (*n* = 132, 19%), reflecting the relative proportion of the different pathogens. Patients received a median of three (IQR: 2–4) different antibiotics.

**Table 3:** Treatment strategies according to microbiological findings

	*Fusobacterium* spp. (*n* = 415)	*Streptococcus/Staphylococcus* spp. (*n* = 108)	Other Bacteria (*n* = 51)	Negative Culture (*n* = 138)	Total (*n* = 712)
**Antibiotics**					
Empiric Antibiotic Treatment	86 (21%)	19 (18%)	9 (18%)	38 (28%)	152 (21%)
Number of antibiotics Median, [Q1, Q3]	3 [2, 4]	3 [2, 4]	3 [2, 4]	2 [2, 3]	3 [2, 4]
Penicillin and aminopenicillines	245 (59%)	56 (52%)	24 (47%)	46 (33%)	371 (52%)
Metronidazole	235 (57%)	30 (28%)	19 (37%)	34 (25%)	318 (45%)
Cephalosporins	166 (40%)	45 (42%)	24 (47%)	44 (32%)	279 (39%)
Clindamycin	145 (35%)	39 (36%)	15 (29%)	26 (19%)	225 (32%)
Carbapenems	90 (22%)	18 (17%)	11 (22%)	13 (9%)	132 (19%)
Vancomycin	56 (14%)	40 (37%)	6 (12%)	8 (6%)	110 (15%)
Quinolones	65 (16%)	7 (7%)	11 (22%)	7 (5%)	90 (13%)
Aminoglycosides	49 (12%)	11 (10%)	9 (18%)	6 (4%)	75 (11%)
**Anticoagulation**					
Prophylactic	23 (6%)	5 (5%)	0 (0%)	3 (2%)	31 (4%)
Therapeutic	164 (40%)	71 (66%)	29 (57%)	74 (54%)	338 (48%)
**Interventions**					
Invasive Treatment	222 (54%)	57 (53%)	33 (65%)	33 (24%)	345 (49%)
Abscess Drainage	169 (41%)	36 (33%)	22 (43%)	25 (18%)	252 (35%)
Lobectomy	2 (1%)	0 (0%)	1 (2%)	0 (0%)	3 (0%)
Mastoidectomy	24 (6%)	6 (6%)	6 (12%)	4 (3%)	40 (6%)
Tonsillectomy	15 (4%)	4 (4%)	1 (2%)	1 (1%)	21 (3%)
Pleural Decortication	11 (3%)	2 (2%)	1 (2%)	0 (0%)	14 (2%)

Overall, 338 (48%) patients received therapeutic anticoagulation: 71 (66%) of the patients with *Streptococcus* spp. or *Staphylococcus* spp. and 164 (40%) with *Fusobacterium* spp. Anticoagulants were prescribed in 29 (57%) and 74 (54%) patients with other bacteria or with negative cultures, respectively.

A total of 345 (49%) patients underwent an invasive procedure. Abscess drainage and mastoidectomy were the most common interventions. Patients presenting with bacteremia other than *Fusobacterium* spp., *Streptococcus* spp. or *Staphylococcus* spp. recorded the highest rate of invasive interventions (*n* = 33, 65% vs. ~53% in the other two groups). Also, the patients infected by other bacteria presented the highest rate of mastoidectomy (12% vs. 6% in the other two groups), possibly reflecting the different genesis of bacterial infection.

We observed a total of 93 (13%) early complications: 34 (5%) patients had a new thrombosis or thrombosis extension, 76 (11%) a peripheral septic lesion, and 19 (3%) a bleeding. No major differences in terms of outcomes were recorded across microbiological groups. Only the subgroup with a negative culture had lower rates of early complications (*n* = 5, 4%) compared to any other microbiological group (Table 4). Overall, late sequelae, such as neurological or orthopedic impairment, were observed in 73 (10%) cases. Again, only the subgroup with negative cultures had substantially lower rates of late sequelae (*n* = 5, 4%), compared with the other groups, in which long-term complications exceeded 11%. A total of 26 (4%) patients died: 15 (4%) of those with *Fusobacterium* spp., six (6%) of those with either *Streptococcus* spp. or *Staphylococcus* spp., three (6%) of those with other bacteria, and two (1%) of those with negative blood cultures.

**Table 4:** Clinical outcomes according to microbiological findings

	*Fusobacterium* spp. (*n* = 415)	*Streptococcus/Staphylococcus* spp. (*n* = 108)	Other (*n* = 51)	Negative Culture (*n* = 138)	Total (*n* = 712)
**Early complications**					
Thrombosis	23 (6%)	7 (7%)	3 (6%)	1 (1%)	34 (5%)
Peripheral septic lesion	54 (13%)	10 (9%)	8 (16%)	4 (3%)	76 (11%)
Bleeding	14 (3%)	3 (3%)	1 (2%)	1 (1%)	19 (3%)
Overall	63 (15%)	15 (14%)	10 (20%)	5 (4%)	93 (13%)
**Late sequelae**	47 (11%)	14 (13%)	7 (14%)	5 (4%)	73 (10%)
**Death**	15 (4%)	6 (6%)	3 (6%)	2 (1%)	26 (4%)

In a sub analysis we examined the characteristics of 16 infections with Enterobacterales and four with non-fermenters, as shown in Tables S1-S3 of the supplementary material. We found that the median age of patients with one of these bacteria was 52 (IQR: 28–61) and 64 (IQR: 32–68) years respectively. Compared with *Fusobacterium* spp., Enterobacterales initially presented with considerably less oropharyngeal infections (*n* = 4, 25%) but with more neck infections (*n* = 9, 56%).

### Sensitivity analysis

In 588 (83%) cases, information on additional cultures from normally sterile body sites different from blood (suppuration, exudates, cerebrospinal fluid) was available. Additional cultures were not performed in 432 cases (73%), were attempted but yielded negative results in 32 (5%) cases, and were performed with positive results in 124 (21%) cases.

Overall, the proportions of patients with *Streptococcus* spp. or *Staphylococcus* spp., and other bacteria were similar between the groups with and without additional cultures from sites other than blood (Table S4, Fig. S1). There was a slight tendency for these proportions to be higher among patients with additional cultures, while the proportion of negative cultures was lower. This observation suggests some degree of contamination in this subgroup, but to an extent unlikely to completely drive the findings of the main analysis.

No significant differences were observed in baseline characteristics, including age distribution (Table S5-S6, Fig. S2-S5). The rate of early complications was higher among patients with additional cultures, which is expected, as new-onset suppurations were included in the definition of early complications, and cultures of sites other than blood were therefore more likely to be performed in those with complications (Table S7, Fig. S6).

## Discussion

Lemierre syndrome is a poorly characterized condition, still associated with high mortality and a considerable burden of complications [2, 3]. Only few case series addressed the disease in the past and none of them systematically focused on its microbiological characteristics [3].

Even though Lemierre syndrome is traditionally associated with the isolation of *Fusobacterium* spp., in our patient-level analysis this agent was found in only 58% of the patients, either alone or in association with other pathogens, primarily with *Streptococcus* spp. and *Staphylococcus* spp [19]. In 21% of the cases, *Streptococcus* spp. or *Staphylococcus* spp. was reported, mostly not combined with *Fusobacterium* spp. Also, a notable fraction of our subjects (19%) showed no bacterial growth and in the remaining 7% various rare aerobes and anaerobes were isolated from cultures but not classified at the species level or observed microscopically. Negative culture results are often reported in Lemierre syndrome and are attributable to two factors. First, empiric antibiotic therapy, initiated in a primary care setting before blood cultures are collected, is common in the early stages of the disease and can result in negative cultures [27]. Second, *Fusobacterium* spp., as many anaerobes, presents challenges for cultivation due to its demanding growth requirements [1, 2, 28, 29].

The finding of a considerable proportion of cases associated with bacteria other than *Fusobacterium* spp., especially *Staphylococcus* spp. or *Streptococcus* spp., is in line with numerous reports of cases clinically indistinguishable from the Lemierre syndrome but associated with these bacteria rather than *Fusobacterium* spp. and substantial preclinical research showed the thrombogenic properties of these microbes [6, 8, 18, 30, 31]. Even under the assumption that *Fusobacterium* spp. was present in these cases but not isolated as it is difficult to cultivate, a synergistical action with *Fusobacterium* spp. cannot be excluded, as demonstrated in vitro [19–21]. These findings raise the question of whether the presence of *Fusobacterium* spp. should remain a strict criterion for diagnosing Lemierre syndrome. Furthermore, it is worth considering the possibility that the strong association of this genus with the disease, as reported in the literature, has been influenced by traditional definitions with requirement of identification of *Fusobacterium* spp., potentially resulting in detection bias and an underestimation of cases associated with other bacterial pathogens [32]. To address these uncertainties, multinational registries and large prospective studies are essential, not only to provide a better understanding of the clinical outcomes of Lemierre syndrome but also to explore the in vivo role of polymicrobial infections and clarify the extent to which coinfections contribute to disease severity. In this context, and in light of the high prevalence of negative cultures seen in reported cases, it is crucial to ensure that diagnostic protocols are as standardized as possible and incorporate advanced microbiological techniques, such as multiplex PCR or MALDI-TOF, to capture a broader spectrum of causative organisms.

The distribution of pathogens we observed may have been affected by some sources of bias, but without a single obvious direction. On the one hand, the traditional definition requiring *Fusobacterium* spp. for a diagnosis is still commonly used and may have led to overestimation of the cases with isolation of this genus among those receiving the diagnosis. On the other hand, the opposite is also possible – cases with bacteria not traditionally associated with the syndrome may have been described more often because of their novelty. Because of the large size of the study population and the restriction to cases reported after 2000s and subjected to peer review, this population may be considered at least representative of the current notion of Lemierre syndrome in the contemporary medical community.

The pathogenesis of Lemierre syndrome has been linked to virulence factors, specific to *Fusobacterium necrophorum*, that facilitate tissue invasion, immune evasion, and possibly thrombophlebitis [13, 33]. Among these, leukotoxin plays a crucial role by targeting neutrophils and macrophages, leading to cell apoptosis and immune suppression. Endotoxin, a lipopolysaccharide common in Gram-negative bacteria, contributes to systemic inflammation. Hemagglutinin has been suggested as a potential factor in the thrombosis characteristic of Lemierre syndrome by promoting platelet aggregation; however, its precise role in the pathogenesis of human infection remains uncertain [13]. Considerable evidence shows that *Staphylococcus aureus* also has strong procoagulant properties, primarily attributed to the staphylocoagulase, which directly induces coagulation by activating prothrombin [31]. Furthermore, *S. aureus* manipulates fibrinolysis by triggering plasminogen activation via staphylokinase, binds and activates platelets, and interacts with key coagulation proteins such as fibrinogen, fibronectin, and von Willebrand factor. Similarly, *Streptococcus* spp. exhibit procoagulant properties through the secretion of SIC (streptococcal inhibitor of complement) protein. SIC binds fibrin via its central region and inhibits fibrinolysis by interacting with plasminogen [18]. Finally, it has been repeatedly hypothesized that *Staphylococcus* spp. and *Streptococcus* spp. may have a synergistic effect with *Fusobacterium* spp. in the pathogenesis of Lemierre syndrome, though further research is needed to clarify this interaction [21]. Infection by the Epstein-Barr Virus mostly affects the same age group as Lemierre syndrome and has been hypothesized to be associated with it, but studies so far have been inconclusive [34, 35]. In our study population as well, the limited extent of testing for Epstein-Barr Virus prevents definitive conclusions. Further research is necessary in this direction.

In the study of pathogen prevalence among various age groups, *Fusobacterium* spp. emerged as the most frequently isolated pathogen in children and young adults. However, its prevalence significantly decreases in individuals over 45 years of age, where *Streptococcus* spp. and *Staphylococcus* spp. become more common. This shift can be attributed to the natural changes in the oral commensal flora that occur from childhood through adolescence. It is notable that anaerobes, including various *Fusobacterium* species, begin colonizing the oral cavity after the first year of life [36, 37]. Among these, *Fusobacterium nucleatum* is typically a component of the normal oral flora, while *Fusobacterium necrophorum* is more often associated with disease [38]. This distinction was evident in a case-control study of outpatients with acute pharyngitis, where *Fusobacterium necrophorum* was isolated in 10% of the cases but was absent in healthy controls and the oldest infected patient was 43 years old [39]. Similarly, a retrospective, population-based surveillance cohort study confirmed that, while *Fusobacterium nucleatum* is the predominant species across a broader age range, *Fusobacterium necrophorum* prevalently infects younger patients without comorbidities and is notably absent in those older than 40 years [40]. The observed variation in pathogen prevalence across age groups may have clinical implications, if confirmed in future prospective studies, particularly for the empiric antibiotic therapy of head and neck infections. For instance, the predominance of *Fusobacterium* spp. in younger patients and the higher prevalence of *Streptococcus* spp. and *Staphylococcus* spp. in older patients may suggest that empiric treatment should ensure adequate anaerobic coverage in the former and a broader spectrum, including Gram-positive organisms, in the latter.

We observed different patterns of bacterial growth depending on the site of primary infection. *Fusobacterium* spp. was associated with oropharyngeal infection in 80% of the cases, while *Streptococcus* spp. or *Staphylococcus* spp. only in 57%. Between 1950s and 1980s antibiotics were commonly used for tonsillitis, which may have led to a decrease in the number of cases of Lemierre syndrome and anaerobic septicaemia with oropharyngeal entry [41]. Later, the introduction of point of care testing (e.g. Rapid Strep Test, RST) and the implementation of guidelines for the antimicrobial management of outpatients led to a more cautious and restrictive use of antibiotics. Some authors have suggested that, in turn, this may have led to a resurgence of the syndrome, with increased incidence [13, 42]. As most cases are associated with sore throat, the age distribution of Lemierre syndrome resembles that of tonsillopharyngitis, which also peaks among young adults. While *Fusobacterium necrophorum* is relatively common in acute tonsillitis, it is even more frequent in chronic or recurrent tonsillitis [43–45].

*Streptococcus* spp. and *Staphylococcus* spp. were associated with a higher prevalence of internal jugular vein thrombosis at presentation, affecting 94% of cases, in contrast to only 63% for *Fusobacterium* spp. On the other hand, *Fusobacterium* spp. was detected in nearly all cases of septic embolism. This disparity could be due to a detection bias, where diagnostic imaging might be more frequently utilized when *Fusobacterium* spp. is suspected, owing to its strong link with septic emboli compared to other bacteria. Additionally, in cases identified as Lemierre syndrome – traditionally characterized in their definition by internal jugular vein thrombosis and/or isolation of *Fusobacterium* spp. – those involving only *Staphylococcus* spp. or *Streptococcus* spp. might not meet the diagnostic criteria for Lemierre syndrome without the presence of internal jugular vein thrombosis. Conversely, cases with *Fusobacterium* spp. but without internal jugular vein thrombosis have been diagnosed with Lemierre syndrome. Hence the spurious correlation between internal jugular vein thrombosis and *Staphylococcus* spp. or *Streptococcus* spp.

While associated to specific presentation patterns, as already observed for differential blood cell count, bacterial species seem not to appear prognostically relevant for in-hospital complications [46].

## Limitations

Our study has several limitations that merit consideration. The data was derived from a systematic review of cases reported by physicians from different centers over an extended inclusion period. While this approach allowed us to collect a large patient population of a rare disease, it had a number of disadvantages that require care when interpreting the results.

First, some degree of reporting bias cannot be excluded. The level of detail provided on diagnostic procedures and testing practices differed across patients, with reports collected by diverse specialists (encompassing emergency medicine, clinical infectious diseases, radiology, surgery, intensive care, family medicine), healthcare settings (primary care, inpatient or outpatient secondary care) and publication forms (case reports, single-center case series, academic theses). To address these sources of variability and reduce bias, in the stage of data collection we adopted the broadest possible definition of the syndrome, avoided language restrictions, included grey literature, and directly contacted authors for missing data or additional cases. However, data remained mostly limited to the information available in a general patient chart in a clinical setting.

Second, the lack of standardization in diagnostic and testing procedures complicates the comparability of the microbiological findings reported by different centers. In particular, the possibility should be considered that some microorganisms identified as pathogens were, in fact, contaminants. Because Lemierre syndrome is associated with a substantial risk of septic embolization with suppurations, the cultured material (blood, suppuration, or other normally sterile bodily fluid) may represent a potential source of bias on the microbiological findings in this condition. To address this issue, we conducted a sensitivity analysis by comparing the bacteria identified in patients in whom only blood cultures were carried out with those identified in patients in whom both blood cultures and cultures from other sites (pus, exudates, or other normally sterile body fluids) were carried out. The analysis showed that, in the second group, *Streptococcus* spp., *Staphylococcus* spp., and other bacteria other than *Fusobacterium* spp. were identified in only a marginally higher proportion than in the first group, showing that while some contamination may have been present, its extent was not such as to determine completely the results of the main analysis. In light of these limitations the interpretation of the findings should primarily be viewed as reflective of the real-world practice and hypothesis-generating rather than aimed at drawing definitive conclusions on pathogenicity.

Last, as in most studies on Lemierre syndrome, blood cultures were negative in a considerable proportion of the patients. This added some uncertainty to the distribution of bacteria we observed.

## Conclusion

We provided the first description of the microbiological epidemiology of Lemierre syndrome with patient-level data. The variety of agents involved in the clinical syndrome suggest greater diversity in its microbiological background than believed until now. This hypothesis should be verified by future registries or prospective studies with standardized methods, as its confirmation may have implications for public health surveillance, diagnostic strategies in acutely ill patients, and empiric antibiotic therapy in primary and secondary care. Variations in the demographic, clinical presentation and initial treatment across patient groups presenting specific pathogens may reflect differences in clinical course across initial infections and etiologic agents, or constraints imposed by traditional, restrictive definitions of the syndrome on the features of the cases reported.

## Electronic supplementary material

Below is the link to the electronic supplementary material.

Supplementary Material 1

## Data Availability

Individual deidentified data will be available following publication on request from the authors. Proposals should be directed to the corresponding author.
