# Supplementary material for: Microbiological diversity among patients with Lemierre syndrome and clinical implications: an individual patient-level analysis
Source: Infection. 2025 Feb 15;53(5):1745–54. doi: 10.1007/s15010-025-02489-w (PMC12460448; doi:10.1007/s15010-025-02489-w)
Supplement: Supplementary file 1 — Supplementary Material 1 [file 15010_2025_2489_MOESM1_ESM.docx]

**SUPPLEMENTARY MATERIAL**

**Table S1. Baseline characteristics stratified by Fusobacterium, Streptococcus/Staphylococcus, non-fermenters (Acinetobacter, Pseudomonas), and Enterobacterales (E. coli, Citrobacter, Enterobacter, Klebsiella, Serratia, Proteus)**

|  | **Fusobacterium spp. (N=415)** | **Streptococcus spp./ Staphylococcus spp.**  **(N=108)** | **Enterobacterales (N=16)** | **Non-Fermenter (N=5)** | **Other Bacteria (N=30)** | **Negative Culture (N=138)** | **Overall (N=712)** |
| --- | --- | --- | --- | --- | --- | --- | --- |
| **Women** | 164 (40%) | 43 (40%) | 8 (50%) | 2 (40%) | 15 (50%) | 63 (46%) | 295 (41%) |
| **Median age [Q1, Q3]** | 20 [16, 27] | 25 [16, 49] | 52 [28, 61] | 64 [32, 68] | 21 [17, 36] | 24 [18, 43] | 21 [17, 33] |
| **Cancer** | 3 (1%) | 4 (4%) | 1 (6%) | 0 (0%) | 0 (0%) | 4 (3%) | 12 (2%) |
| **Site of Infection** |  |  |  |  |  |  |  |
| **Oropharyngeal Infection** | 333 (80%) | 62 (57%) | 4 (25%) | 1 (20%) | 17 (57%) | 103 (75%) | 520 (73%) |
| **LRT Infection** | 214 (52%) | 36 (33%) | 6 (38%) | 0 (0%) | 15 (50%) | 59 (43%) | 330 (46%) |
| **Neck Infection** | 136 (33%) | 52 (48%) | 9 (56%) | 0 (0%) | 14 (47%) | 76 (55%) | 287 (40%) |
| **Ear Infection** | 45 (11%) | 12 (11%) | 2 (13%) | 2 (40%) | 7 (23%) | 16 (12%) | 84 (12%) |
| **Dental Infection** | 26 (6%) | 16 (15%) | 2 (13%) | 0 (0%) | 6 (20%) | 10 (7%) | 60 (8%) |
| **Clinical Presentation** |  |  |  |  |  |  |  |
| **IJV Thrombosis** | 261 (63%) | 101 (94%) | 16 (100%) | 4 (80%) | 25 (83%) | 119 (86%) | 526 (74%) |
| **Cerebral Vein Thrombosis** | 63 (15%) | 38 (35%) | 6 (38%) | 2 (40%) | 6 (20%) | 28 (20%) | 143 (20%) |
| **EJV Thrombosis** | 20 (5%) | 9 (8%) | 2 (13%) | 0 (0%) | 1 (3%) | 11 (8%) | 43 (6%) |
| **Septic Embolism** | 377 (91%) | 74 (69%) | 11 (69%) | 3 (60%) | 27 (90%) | 90 (65%) | 582 (82%) |
| **Pulmonary Septic Embolism** | 326 (79%) | 65 (60%) | 8 (50%) | 1 (20%) | 24 (80%) | 82 (59%) | 506 (71%) |
| **JBM Septic Embolism** | 72 (17%) | 18 (17%) | 3 (19%) | 0 (0%) | 4 (13%) | 11 (8%) | 108 (15%) |
| **Intracranial Septic Embolism** | 52 (13%) | 13 (12%) | 0 (0%) | 3 (60%) | 2 (7%) | 9 (7%) | 79 (11%) |
| **Liver Septic Embolism** | 9 (2%) | 1 (1%) | 0 (0%) | 0 (0%) | 2 (7%) | 2 (1%) | 14 (2%) |

LRT = lower respiratory tract, IJV = internal jugular vein, EJV = external jugular vein, JBM = joints, bone or muscle

**Table S2. Age classes stratified by Fusobacterium, Streptococcus/Staphylococcus, non-fermenters (Acinetobacter, Pseudomonas), and Enterobacterales (E. coli, Citrobacter, Enterobacter, Klebsiella, Serratia, Proteus)**

|  | **0-15**  **(N=128)** | **16-30**  **(N=385)** | **31-45**  **(N=99)** | **>45**  **(N=99)** | **Overall (N=711)** |
| --- | --- | --- | --- | --- | --- |
| **Fusobacterium spp.** | 78 (61%) | 265 (69%) | 51 (52%) | 21 (21%) | 415 (58%) |
| **Streptococcus spp./Staphylococcus spp.** | 26 (20%) | 35 (9%) | 17 (17%) | 30 (30%) | 108 (15%) |
| **Enterobacterales** | 1 (1%) | 4 (1%) | 1 (1%) | 10 (10%) | 16 (2%) |
| **Non-Fermenter** | 0 (0%) | 1 (0%) | 1 (1%) | 3 (3%) | 5 (1%) |
| **Other Bacteria** | 5 (4%) | 14 (4%) | 5 (5%) | 6 (6%) | 30 (4%) |
| **Negative Culture** | 18 (14%) | 66 (17%) | 24 (24%) | 29 (29%) | 137 (19%) |

**Table S3. Outcomes stratified by Fusobacterium, Streptococcus/Staphylococcus, non-fermenters (Acinetobacter, Pseudomonas), and Enterobacterales (E. coli, Citrobacter, Enterobacter, Klebsiella, Serratia, Proteus)**

|  | ***Fusobacterium* spp. (n = 415)** | ***Streptococcus*/ *Staphylococcus* spp.**  **(n = 108)** | **Enterobacterales (N=16)** | **Non-Fermenter (N=5)** | **Other Bacteria (N=30)** | **Negative Culture (n = 138)** | **Total (n = 712)** |
| --- | --- | --- | --- | --- | --- | --- | --- |
| **Early complications** |  |  |  |  |  |  |  |
| **Thrombosis** | 23 (6%) | 7 (7%) | 4 (25%) | 1 (20%) | 5 (17%) | 1 (1%) | 34 (5%) |
| **Peripheral septic lesion** | 54 (13%) | 10 (9%) | 1 (6%) | 0 (0%) | 2 (7%) | 4 (3%) | 76 (11%) |
| **Bleeding** | 14 (3%) | 3 (3%) | 3 (19%) | 1 (20%) | 4 (13%) | 1 (1%) | 19 (3%) |
| **Overall** | 63 (15%) | 15 (14%) | 0 (0%) | 0 (0%) | 1 (3%) | 5 (4%) | 93 (13%) |
| **Late sequelae** | 47 (11%) | 14 (13%) | 3 (19%) | 1 (20%) | 3 (10%) | 5 (4%) | 73 (10%) |
| **Death** | 15 (4%) | 6 (6%) | 1 (6.3%) | 0 (0%) | 2 (7%) | 2 (1%) | 26 (4%) |

**Table S4. Distribution of bacteria across patients with vs without additional cultures on pus or exudates**

|  | **No additional cultures (N = 432)** | **Additional cultures (N = 156)** |
| --- | --- | --- |
| **Fusobacterium spp.** | 263 (61%) | 96 (62%) |
| **Negative Culture** | 84 (19%) | 13 (8.3%) |
| **Other Bacteria** | 25 (6%) | 19 (12%) |
| **Streptococcus spp./Staphylococcus spp.** | 60 (14%) | 28 (18%) |

**Table S5. Baseline characteristics and early complications across patients with vs without additional cultures on pus or exudates**

|  | **NO ADDITIONAL CULTURES** | | | | | **ADDITIONAL CULTURES** | | | | |
| --- | --- | --- | --- | --- | --- | --- | --- | --- | --- | --- |
|  | ***Fusobacterium* spp.**  **(N=263)** | **Negative Culture**  **(N=84)** | **Other Bacteria**  **(N=25)** | ***Streptococcus* spp./ *Staphylococcus* spp.**  **(N=60)** | **Overall**  **(N=432)** | ***Fusobacterium* spp.**  **(N=96)** | **Negative Culture**  **(N=13)** | **Other Bacteria**  **(N=19)** | ***Streptococcus* spp./ *Staphylococcus* spp.**  **(N=28)** | **Overall**  **(N=156)** |
| **Women** | 107 (40.7%) | 35 (41.7%) | 13 (52.0%) | 30 (50.0%) | 185 (42.8%) | 33 (34.4%) | 9 (69.2%) | 10 (52.6%) | 6 (21.4%) | 58 (37.2%) |
| **Median age [Q1, Q3]** | 21.0 [17.0, 27.0] | 23.0 [18.0, 42.0] | 24.0 [19.0, 50.0] | 23.5 [16.0, 44.3] | 21.0 [17.0, 31.5] | 19.0 [15.0, 26.0] | 21.0 [13.0, 31.0] | 21.0 [17.0, 54.5] | 24.0 [16.0, 35.3] | 20.0 [15.0, 29.3] |
| **Age groups** | | | | | | | | | | |
| **0-15** | 41 (15.6%) | 9 (10.7%) | 2 (8.0%) | 14 (23.3%) | 66 (15.3%) | 26 (27.1%) | 4 (30.8%) | 4 (21.1%) | 7 (25.0%) | 41 (26.3%) |
| **16-30** | 174 (66.2%) | 41 (48.8%) | 12 (48.0%) | 23 (38.3%) | 250 (57.9%) | 55 (57.3%) | 5 (38.5%) | 6 (31.6%) | 11 (39.3%) | 77 (49.4%) |
| **31-45** | 35 (13.3%) | 18 (21.4%) | 4 (16.0%) | 9 (15.0%) | 66 (15.3%) | 9 (9.4%) | 2 (15.4%) | 1 (5.3%) | 6 (21.4%) | 18 (11.5%) |
| **>45** | 13 (4.9%) | 15 (17.9%) | 7 (28.0%) | 14 (23.3%) | 49 (11.3%) | 6 (6.3%) | 2 (15.4%) | 8 (42.1%) | 4 (14.3%) | 20 (12.8%) |
| **Cancer** | 3 (1.1%) | 2 (2.4%) | 0 (0%) | 1 (1.7%) | 6 (1.4%) | 0 (0%) | 0 (0%) | 0 (0%) | 1 (3.6%) | 1 (0.6%) |
| **Oropharyngeal Infection** | 217 (82.5%) | 63 (75.0%) | 12 (48.0%) | 42 (70.0%) | 334 (77.3%) | 69 (71.9%) | 7 (53.8%) | 6 (31.6%) | 14 (50.0%) | 96 (61.5%) |
| **LRT Infection** | 155 (58.9%) | 36 (42.9%) | 15 (60.0%) | 22 (36.7%) | 228 (52.8%) | 26 (27.1%) | 4 (30.8%) | 3 (15.8%) | 10 (35.7%) | 43 (27.6%) |
| **Neck Infection** | 108 (41.1%) | 57 (67.9%) | 12 (48.0%) | 33 (55.0%) | 210 (48.6%) | 24 (25.0%) | 6 (46.2%) | 9 (47.4%) | 15 (53.6%) | 54 (34.6%) |
| **Ear Infection** | 20 (7.6%) | 7 (8.3%) | 6 (24.0%) | 4 (6.7%) | 37 (8.6%) | 19 (19.8%) | 5 (38.5%) | 4 (21.1%) | 2 (7.1%) | 30 (19.2%) |
| **Dental Infection** | 19 (7.2%) | 7 (8.3%) | 6 (24.0%) | 9 (15.0%) | 41 (9.5%) | 7 (7.3%) | 1 (7.7%) | 2 (10.5%) | 7 (25.0%) | 17 (10.9%) |
| **Clinical presentation** | | | | | | | | | | |
| **IJV Thrombosis** | 191 (72.6%) | 73 (86.9%) | 20 (80.0%) | 57 (95.0%) | 341 (78.9%) | 45 (46.9%) | 10 (76.9%) | 18 (94.7%) | 26 (92.9%) | 99 (63.5%) |
| **Cerebral Vein Thrombosis** | 30 (11.4%) | 16 (19.0%) | 5 (20.0%) | 18 (30.0%) | 69 (16.0%) | 26 (27.1%) | 5 (38.5%) | 9 (47.4%) | 10 (35.7%) | 50 (32.1%) |
| **EJV Thrombosis** | 18 (6.8%) | 6 (7.1%) | 1 (4.0%) | 6 (10.0%) | 31 (7.2%) | 2 (2.1%) | 0 (0%) | 2 (10.5%) | 2 (7.1%) | 6 (3.8%) |
| **Septic Embolism** | 244 (92.8%) | 57 (67.9%) | 22 (88.0%) | 45 (75.0%) | 368 (85.2%) | 83 (86.5%) | 6 (46.2%) | 15 (78.9%) | 23 (82.1%) | 127 (81.4%) |
| **Pulmonary Septic Embolism** | 220 (83.7%) | 52 (61.9%) | 19 (76.0%) | 39 (65.0%) | 330 (76.4%) | 60 (62.5%) | 6 (46.2%) | 11 (57.9%) | 22 (78.6%) | 99 (63.5%) |
| **JBM Septic Embolism** | 31 (11.8%) | 7 (8.3%) | 3 (12.0%) | 11 (18.3%) | 52 (12.0%) | 35 (36.5%) | 2 (15.4%) | 4 (21.1%) | 5 (17.9%) | 46 (29.5%) |
| **Intracranial Septic Embolism** | 26 (9.9%) | 5 (6.0%) | 2 (8.0%) | 8 (13.3%) | 41 (9.5%) | 20 (20.8%) | 1 (7.7%) | 3 (15.8%) | 4 (14.3%) | 28 (17.9%) |
| **Liver Septic Embolism** | 7 (2.7%) | 1 (1.2%) | 1 (4.0%) | 0 (0%) | 9 (2.1%) | 2 (2.1%) | 0 (0%) | 1 (5.3%) | 0 (0%) | 3 (1.9%) |

LRT = lower respiratory tract, IJV = internal jugular vein, EJVT = external jugular vein, JBM = joints, bones or muscle

**Table S6. Age distribution across patients with vs without additional cultures on pus or exudates**

|  | **NO ADDITIONAL CULTURES** | | | | | **ADDITIONAL CULTURES** | | | | |
| --- | --- | --- | --- | --- | --- | --- | --- | --- | --- | --- |
|  | **0-15**  **(N=66)** | **16-30**  **(N=250)** | **31-45**  **(N=66)** | **>45**  **(N=49)** | **Overall**  **(N=431)** | **0-15**  **(N=41)** | **16-30**  **(N=77)** | **31-45**  **(N=18)** | **>45**  **(N=20)** | **Overall**  **(N=156)** |
| ***Fusobacterium* spp.** | 41 (62.1%) | 174 (69.6%) | 35 (53.0%) | 13 (26.5%) | 263 (61.0%) | 26 (63.4%) | 55 (71.4%) | 9 (50.0%) | 6 (30.0%) | 96 (61.5%) |
| **Negative Culture** | 9 (13.6%) | 41 (16.4%) | 18 (27.3%) | 15 (30.6%) | 83 (19.3%) | 4 (9.8%) | 5 (6.5%) | 2 (11.1%) | 2 (10.0%) | 13 (8.3%) |
| **Other Bacteria** | 2 (3.0%) | 12 (4.8%) | 4 (6.1%) | 7 (14.3%) | 25 (5.8%) | 4 (9.8%) | 6 (7.8%) | 1 (5.6%) | 8 (40.0%) | 19 (12.2%) |
| ***Streptococcus* spp. /**  ***Staphylococcus* spp.** | 14 (21.2%) | 23 (9.2%) | 9 (13.6%) | 14 (28.6%) | 60 (13.9%) | 7 (17.1%) | 11 (14.3%) | 6 (33.3%) | 4 (20.0%) | 28 (17.9%) |

**Table S7. Early outcomes across patients with vs without additional cultures on pus or exudates**

|  | **NO PUS CULTURES** | | | | | **PUS CULTURES** | | | | |
| --- | --- | --- | --- | --- | --- | --- | --- | --- | --- | --- |
|  | ***Fusobacterium* spp.**  **(N=263)** | **Negative Culture**  **(N=84)** | **Other Bacteria**  **(N=25)** | ***Streptococcus* spp./ *Staphylococcus* spp.**  **(N=60)** | **Overall**  **(N=432)** | ***Fusobacterium* spp.**  **(N=96)** | **Negative Culture**  **(N=13)** | **Other Bacteria**  **(N=19)** | ***Streptococcus* spp./ *Staphylococcus* spp.**  **(N=28)** | **Overall**  **(N=156)** |
| **Early complications** | | | | | | | | | | |
| **Thrombosis** | 16 (6.1%) | 1 (1.2%) | 1 (4.0%) | 2 (3.3%) | 20 (4.6%) | 6 (6.3%) | 0 (0%) | 1 (5.3%) | 4 (14.3%) | 11 (7.1%) |
| **Peripheral septic lesion** | 32 (12.2%) | 4 (4.8%) | 1 (4.0%) | 4 (6.7%) | 41 (9.5%) | 18 (18.8%) | 0 (0%) | 6 (31.6%) | 4 (14.3%) | 28 (17.9%) |
| **Bleeding** | 8 (3.0%) | 1 (1.2%) | 0 (0%) | 1 (1.7%) | 10 (2.3%) | 6 (6.3%) | 0 (0%) | 1 (5.3%) | 2 (7.1%) | 9 (5.8%) |
| **Overall** | 38 (14.4%) | 5 (6.0%) | 2 (8.0%) | 5 (8.3%) | 50 (11.6%) | 20 (20.8%) | 0 (0%) | 6 (31.6%) | 6 (21.4%) | 32 (20.5%) |
| **Death** | 7 (2.7%) | 2 (2.4%) | 0 (0%) | 2 (3.3%) | 11 (2.5%) | 7 (7.3%) | 0 (0%) | 2 (10.5%) | 3 (10.7%) | 12 (7.7%) |

**Fig. S1. Distribution of microbiological findings across patients with vs without additional cultures on pus or exudates**

**Fig. S2 Age distribution across patients with vs without additional cultures on pus or exudates**

**Fig. S3. Prevalence of antecedent lower respiratory tract infection across patients with vs without additional cultures on pus or exudates**

**Fig. S4. Prevalence of Antecedent oropharyngeal infection across patients with vs without additional cultures on pus or exudates**

**Fig. S5. Prevalence of septic embolism at presentation across patients with vs without additional cultures on pus or exudates**

**Fig. S6. Prevalence of early complications (thrombosis, septic embolism, bleeding) across patients with vs without additional cultures on pus or exudates**
